# DISCUSSING THE FUTURE FOLLOWING A DIAGNOSIS OF DEMENTIA. FINDINGS FROM THE PREPARED STUDY (ENGLAND)

**DOI:** 10.1093/geroni/igad104.1779

**Published:** 2023-12-21

**Authors:** Josie Dixon

**Affiliations:** London School of Economics and Political Science, London, England, United Kingdom

## Abstract

The PrepareD study (England) adopts a relational autonomy perspective to examine the various conversations that people with dementia and their families have about the future, with each other and with health and care providers, in the year following a dementia diagnosis. We present survey data gathered from a large cohort of 900 people in England who have been recently diagnosed with dementia and their caregivers about informal conversations about the future and advance care planning conversations with health and care providers. We also present findings from in-depth qualitative research with 30 of these respondents, a purposively selected mix of people with dementia and caregivers. Findings from the qualitative research cover how people with dementia and family members interact; the objectives, hopes and expectations they have for their conversations; and how input from professionals and wider social influences shape these interactions. We use findings from the study to reflect on what effective support for informal conversations between people newly diagnosed with dementia and their families about future care could look like and how, and by whom, it could be provided. We also aim to identify theoretical models of relational autonomy that could be useful for developing these approaches further.

